# Proteolytic Regulation of Parathyroid Hormone-Related Protein: Functional Implications for Skeletal Malignancy

**DOI:** 10.3390/ijms20112814

**Published:** 2019-06-08

**Authors:** Jeremy S. Frieling, Conor C. Lynch

**Affiliations:** Tumor Biology Department, H. Lee Moffitt Cancer Center and Research Institute, Tampa, FL 33612, USA; jeremy.frieling@moffitt.org

**Keywords:** PTHrP, proteases, bone, cancer, parathyroid hormone-related protein, metalloproteases, bone metastasis, osteoporosis

## Abstract

Parathyroid hormone-related protein (PTHrP), with isoforms ranging from 139 to 173 amino acids, has long been implicated in the development and regulation of multiple tissues, including that of the skeleton, via paracrine and autocrine signaling. PTHrP is also known as a potent mediator of cancer-induced bone disease, contributing to a vicious cycle between tumor cells and the bone microenvironment that drives the formation and progression of metastatic lesions. The abundance of roles ascribed to PTHrP have largely been attributed to the N-terminal 1–36 amino acid region, however, activities for mid-region and C-terminal products as well as additional shorter N-terminal species have also been described. Studies of the protein sequence have indicated that PTHrP is susceptible to post-translational proteolytic cleavage by multiple classes of proteases with emerging evidence pointing to novel functional roles for these PTHrP products in regulating cell behavior in homeostatic and pathological contexts. As a consequence, PTHrP products are also being explored as potential biomarkers of disease. Taken together, our enhanced understanding of the post-translational regulation of PTHrP bioactivity could assist in developing new therapeutic approaches that can effectively treat skeletal malignancies.

## 1. Introduction

Thirty years have passed since the discovery of parathyroid hormone-related protein (PTHrP) as a tumor-derived hormone responsible for humoral hypercalcemia of malignancy (HHM) [1]. Subsequent to this discovery, several potent effects of PTHrP in bone biology and cancer-induced bone disease have been identified. Elegant in vivo studies using genetically engineered mice have exposed vital roles for PTHrP beginning during embryogenesis and extending into adult life, suggesting that PTHrP has potent, multi-functional effects. 

PTHrP is a member of the parathyroid family of hormones. The PTHrP gene, *PTHLH,* is located on the short arm of chromosome 12 whereas the gene for parathyroid hormone (*PTH*) itself is found on chromosome 11 [2,3]. The resulting protein is largely conserved among most species, however, alternative splicing produces three unique isoforms of human PTHrP (139, 141, or 173 amino acids) that are differentially expressed in specific tissues [3]. The precise purposes and characterization of these isoforms have not been elucidated at this juncture. However, the presence of varying instability motifs between the mRNA isoforms suggests that each possess distinct half-lives and that some may be more suitable to functioning as a paracrine or autocrine mediator as opposed to the more conventional endocrine activities associated with PTH [4]. 

As would be expected, the PTHrP protein shares homology with PTH, but this occurs primarily in the N-terminal region, where eight of the initial 13 amino acid residues (Val2, Ser3, Glx4, Gln6, Leu7, His9, Gly12, and Lys13) are identical [5]. The remainder of the amino acid sequences show little homology [2]. These similarities contribute to PTHrP and PTH activating a common signaling receptor, the type I PTH receptor (PTH1R) [4]. Likewise, the differences in sequence, particularly His5, provide discrimination between receptors. This is observed in the case of PTH2R, which PTH activates whereas PTHrP does not [6]. 

**Figure 1:**
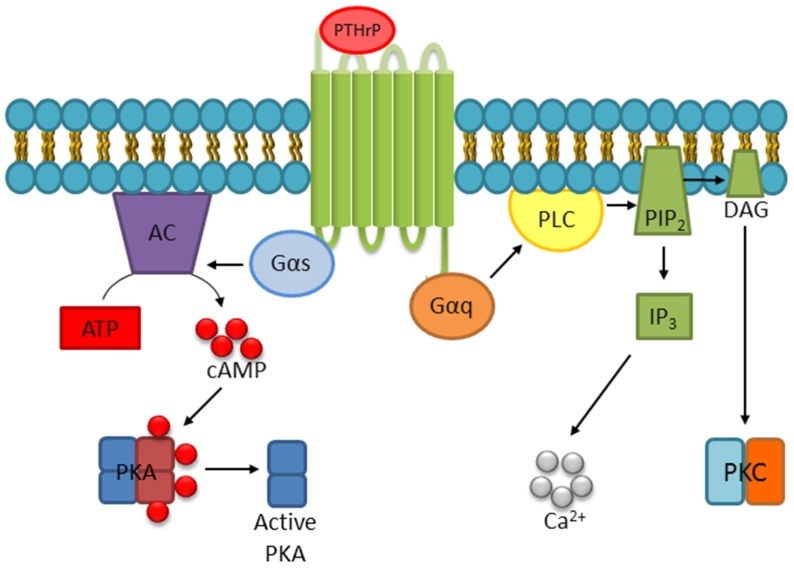
Traditional parathyroid hormone-related protein (PTHrP) activities are mediated through a G-protein coupled receptor (GPCR) called PTHR1. In skeletal tissues, PTHR1 is expressed on the surface of osteoblasts, osteocytes, and chondrocytes. The downstream pathways consist of signaling arms capable of activating protein kinase A (PKA) or protein kinase C (PKC).

PTH1R is a G-protein coupled receptor that predominantly signals through G_S_ alpha subunit (Gα_S_)/adenylyl cyclase/cyclic adenosine monophosphate (cAMP)/protein kinase A (PKA) and less robustly through G_Q_ alpha subunit (Gα_Q_)/phospholipase C (PLC)/protein kinase C (PKC) signaling cascades to mediate gene transcription and cell fate (Figure 1). Upon ligation, a series of conformational changes in PTH1R lead to a shift of transmembrane domain 3 away from transmembrane domain 6, permitting access to the cytoplasmic loops by G proteins that are associated with the adenylyl cyclase and PLC pathways [7]. Through these signaling pathways, PTHrP stimulates the accumulation of intracellular second messengers such as cAMP, diacylglycerol (DAG), and inositol triphosphate (IP3) which subsequently leads to activation of PKA, PKC, and release of intracellular Ca^2+^, respectively (Figure 1) [8]. This can have further downstream effects including cAMP response-element binding protein (CREB) and extracellular signal-regulated kinases (ERK1/2) phosphorylation [4,9,10]. PTHrP has also been shown to stimulate phospholipase D via a mechanism transduced by Gα_12_ and Gα_13_ through Ras homolog gene family, member A (RhoA) [11,12,13]. Furthermore, studies have demonstrated that PTHrP can activate ERK1/2 following intracellular internalization of PTH1R, dependent on beta-arrestins [14,15,16].

PTHrP contains a leader sequence of 36 amino acids (-36 to -1 signal peptide) utilized for intracellular trafficking and secretion [17]. The leader sequence is typically removed as the nascent peptide enters the rough endoplasmic reticulum [18]. After removal of the leader sequence, the resulting product is considered “pro-PTHrP” and is subject to further post-translational modification and activation by proteolytic cleavage. 

### 1.1. PTHrP in Development and Skeletal Biology 

Unlike PTH, whose expression is restricted to the parathyroid glands, PTHrP is ubiquitously expressed throughout many tissues, including heart, skin, bone marrow, fetal liver, gastric mucosa, adrenal, thyroid, breast, and parathyroid glands that can act in a paracrine, autocrine, and intracrine manner [19,20]. *PTHLH* is transcriptionally regulated via regulatory regions and response elements within its promoter. Vitamin D3/Vitamin D receptor, CREB, Ku antigen, Ets1, Tax1, and SP1 have all been identified as factors that may either increase or decrease PTHrP expression [4,21,22,23,24,25]. These factors themselves are controlled by several cytokines and hormones that have been directly linked to *PTHLH* regulation, including transforming growth factor beta (TGFβ), epidermal growth factor (EGF), and Vitamin D3 [26,27,28,29,30,31]. PTHrP can also be regulated post-transcriptionally by micro RNAs (miRNA) [32]. 

Gene ablation studies in vivo produce phenotypes that revealed a particular importance for PTHrP in skeletal and mammary gland development. Systemic deletion of PTHrP (*Pthlh^-/-^*) yielded a neonatal lethal phenotype, with newborns dying within 24 h of birth due to respiratory failure attributed to defective rib cage formation [33]. These mice also exhibited domed skulls, shortened snouts and mandibles, and short limbs, suggesting specific relevance for PTHrP in endochondral bone formation [33]. Due to neonatal lethality, mice in which PTHrP was delivered or overexpressed in specific tissues were generated, thereby allowing for the analysis of PTHrP in various cell types [34,35]. For example, rescue of PTHrP knockout mice from neonatal death via targeted transgenic expression of PTHrP in chondrocytes revealed a failure of early ductal development and provides evidence of a role for PTHrP in branching morphogenesis in mammary tissues [34]. Further, these mice also display dwarfing and failed tooth eruption [34,35,36]. Consistent with these findings, PTHrP haploinsufficiency produces mice that appear normal at birth but display low bone mass, decreased trabecular thickness and connectivity, and increased adiposity as they approach three months of age [37]. In accord with the mesenchymal phenotypes described by other studies, it has also been established that PTHrP is critical for regulating growth plate development by controlling the proliferation and differentiation of chondrocytes [38,39]. 

Throughout adult life, PTHrP remains an important mediator of skeletal remodeling. It is important to note that PTHrP has a bimodal effect on the skeleton, acting primarily on osteoblasts while indirectly influencing osteoclast activity via cytokines such as receptor activator of nuclear factor kappa beta ligand (RANKL) [40]. As a potent mediator of bone metabolism, PTHrP has been the focus for potential therapeutic agents for disorders such as osteoporosis [4]. These studies have shown that PTHrP dosing and level of exposure are critical for the balance between anabolic and catabolic activity, with intermittent dosing regiments being key to generating an osteogenic response [41,42,43]. Recently, an anabolic PTHrP analog called abaloparatide underwent clinical investigation and FDA approval for osteoporosis (NCT01343004, NCT0167462, NCT00542425). The results of phase III clinical trials showed that treatment of postmenopausal women with abaloparatide for 24 weeks with 40 or 80 µg/kg/day resulted in increases in bone mineral density compared to placebo [4,44,45,46]. Furthermore, a review of the number needed to treat (NNT), a measure of benefit based on the number of patients needed to treat to prevent one additional fracture, indicated that the NNT for abaloparatide was lower than teriparatide in all types of fractures assessed (vertebral, non-vertebral, clinical, and major osteoporotic) [47]. 

### 1.2. PTHrP in Cancer 

The PTHrP protein was initially identified and purified from lung cancer cells [1], and many studies have focused on the role of PTHrP in various malignancies. PTHrP is expressed in numerous cancers where it has often been shown to play contributory roles in progression and metastasis [3]. For example, analyses of PTHrP expression in breast cancer specimens indicate that 60% of primary breast tumors and 90% of bone metastatic breast cancers express PTHrP, suggesting the importance of PTHrP for metastatic progression in bone [48,49]. Bone metastatic breast cancers typically generate osteolytic lesions that are a product of increased osteoclast formation and activity [50]. Traditionally, PTHrP has been associated with driving the osteolytic phenotype by inducing RANKL signaling in osteoblasts, which in turn leads to the formation of bone resorbing osteoblasts [51]. For example, the administration of monoclonal PTHrP neutralizing antibodies in mice inoculated with MDA-MB-231 cells led to a significant reduction in osteolytic bone lesions as well as a decrease in tumor size compared to controls [52]. PTHrP expression is also involved in malignancies that induce osteogenic metastatic lesions, such as prostate cancer [53]. Despite this key difference in disease pathology, PTHrP is also a vital factor in metastatic prostate cancer progression, contributing to pathological bone remodeling and facilitating tumor growth in vivo [54]. It is possible that post-translational processing of PTHrP may contribute to the differential activities observed in these cancers, but to date they remain poorly understood.

While PTHrP proteolysis can be a means by which to dampen the activity of the hormone, evidence suggests that PTHrP products retain key activities. In this review, we will dissect the significance of proteolytic processing related to PTHrP biology, with particular emphasis on roles in bone homeostasis and skeletal malignancy.

### 1.3. PTHrP Protein Structure and Susceptibility to Cleavage

**Figure 2:**
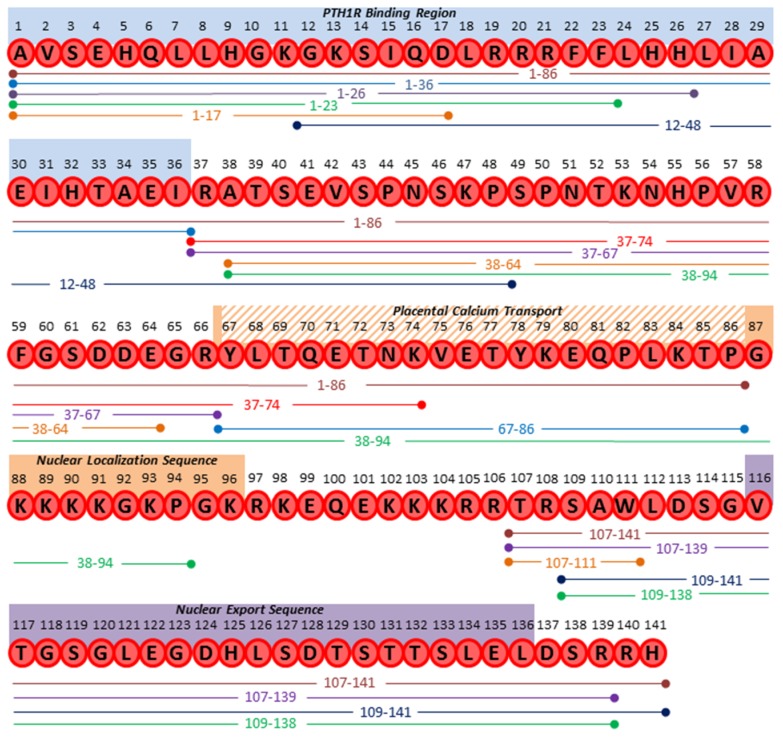
Multiple residues within the PTHrP sequence serve as putative sites for proteolytic cleavage. Currently known PTHrP fragments are shown beneath the amino acid sequence, demonstrating the presence of common residues involved in the generation of these fragments. Functional protein domains are illustrated above the sequence, including the PTH1R binding region (aa. 1–36, blue), placental calcium transport region (aa. 67–86, orange stripes), nuclear localization sequence (aa. 67–96, orange), and nuclear export sequence (aa. 116–136, purple).

While a multitude of functional activities for PTHrP have been reported and are well accepted, it has also been suggested that the full length PTHrP protein (139, 141, or 173 amino acids) may actually serve as a precursor protein that in addition to PTHrP1–36, can be processed into smaller, bioactive proteins (Figure 2) [55]. The presence of multiple predicted mono- and multi-basic cleavage sites within the PTHrP protein sequence indicates that it is likely susceptible to proteolytic cleavage [55]. Indeed removal of the leader sequence and cleavage at Arg37 by enzymes such as furin or prothiol hormone protease have been reported to generate PTHrP1–36 [56,57], therefore, it is possible that additional functions for PTHrP products outside of those known for PTHrP1–36 in bone or other tissues remain to be identified. Growing evidence indicates that subsequent to removal of the leader sequence (-36 to -1), the protein remains subject to further modification by proteolytic cleavage [18]. While the exact cleavage sites and responsible proteases have not been fully elucidated, the susceptibility of PTHrP (full length or other known products) to proteolysis may serve as a mechanism to control the length of exposure and shift PTHrP activity to more paracrine and autocrine roles, as opposed to the canonical endocrine roles ascribed to PTH. Alternatively, this processing may be responsible for the generation of entirely new PTHrP-derived peptide products with novel activities or serve to negatively regulate or dampen PTHrP activities. 

Numerous PTHrP peptide fragments generated by proteolysis have been detected using a combination of in vitro and in vivo approaches, but how these fragment products are generated remains unknown. For example, what are the unique biological functions of the generated peptide products? Does the processing occur intracellularly and/or extracellularly? Are these tissue or cell specific events? Are peptide products further catabolized in a temporal fashion?

## 2. Proteolytic PTHrP Products

Traditionally, most studies have reported that PTHrP1–36 (or 1–34) serves as the mature form of PTHrP, with cleavage at an arginine residue at amino acid position 37 reportedly leading to the secreted PTHrP1–36 [18,56]. This span of 36 amino acids is also relatively homologous to the 34 amino acid sequence of PTH, with eight of the first 13 N-terminal amino acids shared in common [5]. Indeed, most of the classic biological activities attributed to PTHrP, such as regulating osteoblast differentiation [58,59] and promoting osteoclast formation by stimulating osteoblasts to produce RANKL [58], are attributed to this 36 amino acid form. However, the exact proteases involved in the generation of PTHrP1–36 remain poorly defined and are likely tissue context dependent. Furthermore, the abundance of dibasic arginine and lysine residues found in the PTHrP sequence and the predicted susceptibility of PTHrP to undergo processing at these sites raises questions about whether PTHrP1–36 is the only product responsible for classical PTHrP functions. Further still, growing evidence demonstrates that peptides can be generated and measured from the remaining C-terminal portion of PTHrP (37–139, 37–141, 37–173) and that they potentially possess unique activities by acting through PTH1R, novel signaling receptors, or via nuclear translocation (Table 1). Therefore, understanding the post-translational modifications of full length PTHrP by proteolysis and the effects of the resulting products could unravel new functions for PTHrP in development, homeostasis, and disease, and subsequently provide beneficial options for treating conditions such as osteoporosis, skeletal malignancy, and bone metastasis. 

### 2.1. N-terminal Derived Peptides

Although PTHrP1–36 is frequently accepted as the primary form of active PTHrP, shorter N-terminal PTHrP peptides within this region have also been detected. For example, the PTHrP1–23 peptide can be generated from PTHrP1–141 by human kallikrein-3/prostate specific antigen (hk3/PSA), a serine protease most frequently associated with prostate cancer as a biomarker [63]. Studies with the resulting 23 amino acid PTHrP product revealed that PSA cleavage abolishes PTHrP-induced cAMP activity (Figure 1) in MC3T3E1 cells, representing a potential prostate-specific mechanism of regulating PTHrP activity. There is also evidence suggesting that PTHrP1–23 stimulates calvarial new bone formation [65]. In addition to PSA, PTHrP1–23 can also be generated by neprilysin, a member of the metalloprotease class of enzymes that depend on the presence of a metal ion in their active site to catalyze proteolysis [64]. Neprilysin can also generate PTHrP1–26, suggesting that metalloproteases may be important players involved in the post-translational regulation of PTHrP activity while also raising questions as to the temporal dynamics leading to production of either PTHrP1–23 or 26. 

Interestingly, longer PTHrP proteins, such as PTHrP1–86 have also been measured at low circulating levels in patients [71]. PTHrP1–86 appears to have functional effects, particularly in skeletal biology, where it was shown to promote osteogenic differentiation of human MSCs while simultaneously inhibiting their adipogenic differentiation [72]. However, whether PTHrP1–86 undergoes further processing to shorter forms in order to mediate these processes, or if it remains intact to modulate these effects is at present unknown.

### 2.2. Mid-region and C-terminal Derived Peptides

As most PTHrP functions have been attributed to its N-terminal residues, peptides derived from the mid-region and C-terminal region (36–139, 36–141, 36–173) represent an interesting opportunity for study as they could provide insight into novel roles for PTHrP in biology. Numerous C-terminal products have already been described, including mid-region PTHrP products such as PTHrP38–94, 38–111, 67–86, and 12–48. These products have been detected in cell culture extracts, media, or patient samples and with distinct biological roles described [4,56,76,80,81]. For example, PTHrP38–111 has been identified in circulation, along with shorter PTHrP peptides terminating at residues 94, 95, and 101 [82]. Of these, PTHrP38–94 appears to be a mature and significant, functional secreted form [78] that can inhibit cell growth and invasion, as well as limit the viability of breast cancer cell lines such as MDA-MB-231. Additionally, attenuated MDA-MB-231 tumorigenesis in vivo was observed in response to PTHrP38–94 treatment [79]. The same study also defined a mechanism of action for PTHrP38–94, where it was shown to translocate to the nucleoplasm and bind to chromatin. This subsequently induces the expression of Bcl-xS, Bad, Rip1, and activated caspase-2, -5, -6, -7, and -8 [79]. 

Drilling further into shorter C-terminal products, PTHrP67–86 has been reported to restrict the growth but enhance the invasion of 8701-BC human breast cancer cells in vitro [83]. Treatment of breast cancer cells with PTHrP67–86 upregulated the expression of several stress related genes, including heat shock factor binding protein-1 (*hsbp1*) and heat shock protein 90 (*hsp-90*), that sequester and inactivate heat shock factor -1 (hsf1), a protein that normally recognizes ETS transcription factor binding sites within gene promoters [83]. Consequently, this resulted in upregulation of *MMP-1*, downregulation of *uPA*, and acquisition of an invasive phenotype in vitro [83]. Notably, this is an opposite effect of that observed with PTHrP38–94, which impaired breast cancer cell invasion [78]. It is also worth noting that PTHrP67–86 promotes calcium transport across the placenta for fetal skeletal development. This was observed in mice homozygous for deletion of PTHrP which show significant reductions in ionized calcium and maternal-fetal calcium gradients. However, when ^45^Ca was administered into pregnant PTHrP^-/-^ mice, calcium accumulation was restored in the fetuses upon co-injection of PTHrP67–86. Interestingly, calcium transport could not be rescued by PTHrP1–34, indicating that this activity is unique to the mid-region fragment and does not rely on the traditional receptor, PTH1R [84]. 

Recently, PTHrP12–48 was discovered in analysis of the plasma proteome of breast cancer patients with bone metastases [74]. Immunohistochemical analyses localized PTHrP12–48 in metastatic tumor cells of bone metastasis specimens, but it was not present in other bone stromal cells such as osteoblasts or osteocytes. Furthermore, tissues that would normally express PTHrP such as placenta and cartilage were also negative for PTHrP12–48, suggesting that the proteolytic generation of PTHrP12–48 may not occur in normal tissues and instead may be tumor-specific [75].

Osteoclasts, which do not express PTH1R, were also found to be positive for PTHrP12–48. Based upon this observation, the ability of osteoclasts to uptake PTHrP12–48 was therefore tested in vitro. Unlike PTHrP1–36, PTHrP12–48 was readily internalized by both differentiated osteoclasts and osteoclast precursors [75]. However, while other PTHrP fragments have been shown to translocate into cells via PTH1R, PTHrP12–48 appears to rely on a noncanonical intracellular translocation sequence spanning positions 19 to 21 to mediate uptake [75,92,93,94,95]. Intriguingly, this internalization decreased numbers of TRAP positive, multinucleated osteoclasts by inducing cleaved caspase 3-mediated apoptosis [75]. It is presently unconfirmed precisely which proteases generate PTHrP12–48. The study indicated that C-terminal processing involves a member of the prolyl oligopeptidase family of serine proteases [75], while other reports have suggested that dipeptidyl peptidase (DPP) could cleave PTHrP between amino acids 48 and 49 [4]. The N-terminal processing may occur at either Lys11 or Asp10, with Lys11 then being removed by aminopeptidase activity in the latter scenario [75].

Adding to the complexity of C-terminal PTHrP product function, PTHrP38–64 can stimulate type II lung cell growth, which may have implications in lung repair. When added exogenously, an increase in the number of proliferating cell nuclear antigen (PCNA) positive cells (proliferation marker) and cells expressing alkaline phosphatase (a type II cell marker) was observed in addition to increased thymidine incorporation. This may be due in part to an influx of inflammatory cells [77]. However, it is presently unclear if this fragment is generated proteolytically in vivo, as these studies relied on exogenous synthetically generated PTHrP38–64. 

Arguably, beyond PTHrP1–36, the most studied PTHrP product is PTHrP107–139, also known as osteostatin [88]. Although the exact proteases and processes involved in generating PTHrP107–139 are not well characterized, osteostatin may represent an important secretory form of PTHrP. Studies focused on the activity of this peptide have consistently demonstrated its ability to inhibit osteoclast activity both in vitro and in vivo. For example, studies using rat osteoclast cultures demonstrated that PTHrP107–139 reduces the number of mononuclear osteoclast precursors [96]. Subsequent studies using an in vivo calvarial injection model where PTHrP107–139 was injected over the periosteum of the right hemicalvariae for 5 days demonstrated a 70% decrease in osteoclast number, 70% decrease in osteoclast perimeter, and 50% decrease in eroded perimeter along with a 40% decrease in both osteoblast number and osteoblast perimeter and no change in osteoid area, implying that the C-terminal region of PTHrP is involved in regulating osteoclast activity [97]. The anti-resorptive activity of PTHrP107–139 appears to be contained predominantly within amino acids 107 to 111 as studies focused solely on this sequence indicate a highly similar activity profile to PTHrP107–139. Furthermore, reports demonstrated that simultaneous daily administration of both PTHrP107–111 and PTHrP1–34 for either 6 or 16 days ablated the bone resorption induced by PTHrP1–34 alone [86]. Some of these effects, however, may not be solely due to osteoclast mediated anti-resorptive effects. For example, both PTHrP1–36 and PTHrP107–139 yielded anabolic effects on the bones of ovariectomized (OVX) mice following subcutaneous administration (5 days per week for 4 or 8 weeks). It was hypothesized that this may be due to a combined anti-resorptive and pro-formation effect, as ex vivo treatment of bone marrow cells derived from OVX mice as well as in vitro treatment of mouse, mesenchymal C3H10T1/2 cells with PTHrP1–36 or PTHrP107–139 enhanced osteogenic differentiation, possibly through the Wnt signaling pathway [98]. Conversely, and adding to the complexity of PTHrP biology, there is evidence where PTHrP107–111 and 107–139 can stimulate osteoclast-like cell formation in mouse bone cell cultures in vitro. The activity of the resulting osteoclasts were tested on dentine slices, where they were able to resorb and form pits [87].

### 2.3. PTHrP Fragments as Biomarkers of Disease

Beyond potential cellular activities for novel PTHrP fragments, their detection in patient tissues and fluids could also present a unique opportunity for evaluation as biomarkers of disease based on their small size and presence in circulation. For instance, a recent study explored the utility of a novel PTHrP12–48 fragment detected in cancer patient plasma as a prognostic marker for bone metastatic breast cancer (Table 1) [74]. Using SELDI-TOF MS, the plasma proteomes of 36 breast cancer patients were interrogated, and it was determined that PTHrP12–48 could identify patients at risk for bone metastases when combined with serum type I collagen N-terminal telopeptide (NTx) measurements, an established clinical indicator of bone resorption [74]. Because the generation of PTHrP12–48 appears to be tumor-specific, PTHrP12–48 possesses good characteristics for continued biomarker development, and these studies are a significant advancement for the field given the paucity of clinically significant biomarkers for skeletal malignancies.

The utility of PTHrP1–86 as a predictive factor has also been investigated. A study comparing 115 healthy individuals to 122 patients with malignant disease demonstrated that even at low levels, PTHrP1–86 in patient blood could be used as a positive indicator of humoral hypercalcemia of malignancy [71]. Additionally, PTHrP37–67 has been detected in pancreatic tumor tissue extracts, and PTHrP37–74 is present in plasma from patients with humoral hypercalcemia of malignancy [67,73]. Although previous studies have reported that PTHrP may not be a useful marker when assessing if cases of PTHrP-related hypercalcemia are malignant or benign [99], these shorter fragments may still possess potential for future biomarker exploration for other pathologies. 

### 2.4. How Do Novel PTHrP Fragments Mediate Their Effects?

PTHrP normally binds to PTH1R via a “two site model,” where an interaction between the C-terminal domain of active PTHrP (amino acids 15–34) and the N-terminal region of the receptor contributes to binding affinity. Despite differing in amino acid sequences beyond amino acid 13, both PTH and PTHrP contain a crucial alpha-helical binding motif within the amino acids 15–34 sequence [100]. The second interaction occurs between the N-terminal domain of PTHrP and the juxtamembrane region of the PTH1R. This interaction is believed to contribute to the induction of signaling [101]. The specificity of different regions of PTHrP toward PTH1R raises an important question about which receptor(s) are involved in mediating the activities of peptides of mid-region or C-terminal origin, such as osteostatin. Experiments analyzing PTHrP fragment activity have addressed this question by using predictive software simulations and structural studies while other approaches have incorporated commercially available inhibitors of PKA and PKC, both of which are common downstream effectors of GPCRs used to transduce PTH1R signaling (Figure 1) [4,8,75]. For example, molecular docking studies using the predicted alpha helical structure of PTHrP12–48 suggest that residues 12–48 could potentially interact with PTH1R in a manner similar to residues 12–34 present in full length PTHrP, however, subsequent functional assays indicated that despite these predictions, PTHrP12–48 was unable to stimulate cAMP activity in human SaOS2 cells at a range of concentrations (from 0.1 to 1000 nM). For osteostatin, it appears that the effects of PTHrP107–139 may occur through PKC activation since the introduction of PKC inhibitors staurosporine or bisindolylmaleimide I resulted in diminished functionality whereas PKA inhibitors such as H89 did not impact the activity of PTHrP107–139 [87,102,103,104,105]. This preferred PKC route of GPCR signaling is consistent with reports indicating that PTHrP107–111 and 107–139 induce a more robust Ca^2+^ response compared to PTHrP1–36. However, while these data implicate PTH1R as the probable receptor, the induction of Ca^2+^ could not be inhibited by a competitive PTH1R antagonist, PTH7–34 [106], therefore an additional pathway or receptor may also be involved. Further still, little is known about the impact of these fragments on other reported PTHrP signaling outputs, such as phospholipase D or beta-arrestin dependent ERK1/2 activation. 

Taken together, such studies raise the possibility that there could be unknown or unidentified receptors, or even non-receptor activities, targeted by novel PTHrP products [3]. One particular family of receptors that has been studied in this regard is the GPCR endothelin receptor family, composed of endothelin-A (ET_A_), and ET_B_. Of note, PTHrP residues 8–11 resemble the endothelin-1 ligand [61]. Unique PTHrP peptides including PTHrP1–16 and PTHrP1–23 have been studied for their potential stimulation of ET_A_ receptors. For example, PTHrP1–16 was shown to stimulate contractility in rat cardiomyocytes in a cAMP dependent manner and that this could be antagonized with an ET_A_ receptor antagonist [61]. It has also been suggested that PTHrP1–23 may activate ET_A_ receptors [107]. However, other studies with PTHrP1–16 and 1–23 have found no agonistic or antagonistic activities using ET_A_ and ET_B_ bioassays, where neither fragment was able to displace bound ET-1 [108]. These differences in findings may be a result of the different cell types studied, and at this juncture, it cannot be ruled out that PTHrP fragments may act via novel receptors. Further, as evidenced by PTHrP38–94 direct binding with chromatin [79] and PTHrP12–48 detection in osteoclast nuclei [75], certain PTHrP fragments may have roles independent of a signaling receptor. For example, PTHrP can be shuttled from the cytoplasm to the nucleus using a nuclear localization sequence that is typically ascribed to residues contained within the 67–94 sequence. Although this sequence may not be solely responsible, the 67–94 region has been shown via crystallization structures to bind the importin β1 nuclear transport factor [4,17,93]. Interestingly, whereas the conventional nuclear trafficking mechanism requires importin β1 to complex with importinα, nuclear transport factor 2, and GDP-bound Ras-related nuclear protein (RanGDP), the nuclear import of PTHrP utilizes a complex of importin β1 and RanGDP [93,109]. Notably, many of the generated PTHrP fragments lack these residues, including PTHrP12–48 [75]. Therefore, it is possible that other regions of the sequence facilitate nuclear import, including the highly conserved triple arginine motif 19–21 [4,93,94]. Alternatively, other reports have suggested receptor mediated routes for nuclear import of PTHrP from the cell surface [60].

It is important to note that the further processing of PTHrP fragments may also confound observations, and there is expanding evidence that peptide derivatives of PTHrP1–36 or 37–139/141/173 possess distinct functions. However, in the majority of these studies, the stability of the peptide derivatives used to treat cells or tissues in vitro or in vivo is unclear, as is the susceptibility of these products to further modification. Therefore, discrepancies between studies could be due to additional proteolytic processing of PTHrP dependent on tissue context and should be considered.

## 3. Proteolytic Control of PTHrP Function

As a vital component of normal physiology, proteases are responsible for processing proteins by hydrolysis of peptide bonds. Evolutionarily, proteases likely arose as a mechanism to catabolize proteins and facilitate the generation of amino acids [110]. However, a vast array of additional functions for proteases in biology have also been elucidated, including their ability to regulate protein function by generating unique, bioactive forms. These aspects of protease activity have opened a new field of study called degradomics [110,111,112]. To date, more than 588 human proteases have been identified, collectively functioning as a “protease web,” and these can be classified based on their catalytic mechanisms [113,114,115]. Of note, many of these proteases can potentially target PTHrP based on predicted cleavage of amino acid sequences (Table 1).

### 3.1. Proprotein Convertases

The pro-protein convertases belong to a family of serine proteases that are often implicated in the proteolysis and activation/inactivation of multiple proteins including hormones, growth factors, receptors, and enzymes. The family consists of nine secretory serine proteases: proprotein convertase 1 (PC1/3), PC2, furin, PC4, PC5, paired basic amino acid cleaving enzyme 4 (PACE4), PC7, subtilisin kexin isozyme 1 (SKI-1), and proprotein convertase subtilisin kexin 9 (PCSK9). Except for SKI-1 and PCSK9, all of these enzymes cleave at the basic amino acids lysine, arginine, and histidine, many of which occur frequently throughout the PTHrP amino acid sequence [55,116]. These enzymes are not only localized in the Golgi apparatus but also within endosomes, in the extracellular matrix, and at the cell surface [117].

Furin has well established roles in processing pro-proteins via the constitutive secretory pathway, the primary pathway utilized for PTHrP secretion, and it has been implicated in the removal of pre-pro regions of both PTH and PTHrP. The highly conserved Arg-Leu-Lys-Arg sequence that falls between pro-PTHrP and PTHrP is a predicted furin cleavage site, and studies have confirmed that PTHrP is a furin substrate [18]. These observations were made by expressing pro-PTHrP in the COS-7 cell line which endogenously express furin, resulting in high levels of PTHrP being secreted into conditioned cell culture medium. Co-transfecting the cells with both pro-PTHrP and an antisense furin cDNA reduced secretion of active PTHrP [18]. Given the ubiquitous tissue distribution and frequent subcellular localization to the Golgi, furin is likely a key enzyme for the intracellular processing and secretion of active PTHrP. 

It is plausible that pro-protein convertases could cleave PTHrP at other sequence locations as well. Kexin, a yeast serine protease orthologous to human subtilisin-like serine proteases, is known to process pro-protein precursors in a manner similar to mammalian species. Previous reports have demonstrated that kexin cleaves PTHrP1–141 at the triple arginine site 19–21. Substitution of a non-basic alanine for arginine at residue 19 reduces the ability of kexin to process PTHrP [118]. Since many mammalian proprotein convertases are known to cleave at basic residues, it raises the question of whether furin might target PTHrP at this triple arginine site and what the subsequent impact on PTHrP activity would be. Kexin has also been shown to cleave PTHrP at amino acids 97, 105, 106, and 108, therefore these could represent further putative furin cleavage sites [118]. Interestingly, cleavage at residue 106 would contribute to generation of the osteostatin peptide PTHrP107–139. 

Of note, furin can also be trafficked via endosomal vesicles that contain substrate proteins, a mechanism that likely expands its substrate repertoire and raises the possibility of PTHrP processing in additional compartments other than the Golgi. Further, many pro-protein convertases can be active at the cell surface or in the extracellular matrix [117]. These endoproteases, including PACE4, PC1/3, and PC2, have been suggested to process PTHrP at various amino acid sites throughout the protein sequence, many of which are unique from that of putative furin sites [4,82]. However, the biological impact, if any, of the resultant products remains to be determined.

### 3.2. Prostate Specific Antigen

PSA is well-known for its expression in prostate tissue [119], with elevated levels typically correlative of prostate cancer burden. Although PSA has been widely adopted as a prostate cancer biomarker, the functional roles for PSA in prostate cancer are not well understood [120,121]. In normal physiology, PSA degrades semenogelin I and II in seminal fluid [122], however it has also been shown to cleave substrates such as fibronectin, laminin, galectin-3, nidogen-1, and insulin-like growth factor binding protein 3 (IGFBP3) [53,121,123,124,125,126]. Cleavage of these substrates could therefore contribute to increased tumor cell invasion, proliferation, apoptosis, and angiogenesis.

Interestingly, PSA has also been shown to process PTHrP, though the precise function of this activity is unclear. Independent studies have reported the ability of PSA to cleave both PTHrP1–34 and PTHrP1–141, resulting in either 22 or 23 amino acid fragments. Functional analyses of these fragments indicate that neither stimulated cAMP in vitro [63,127], and PSA cleavage may instead serve as a negative regulator of the PTH1R/cAMP mediated activities of PTHrP via degradation of the protein. Alternatively, the implications of this cleavage could be especially interesting considering the heightened expression of both PSA and PTHrP in bone metastatic prostate cancer. It is tempting to speculate that PSA alters PTHrP activity during bone metastasis to prevent the bone resorptive actions of the full length protein but more research is needed to test this hypothesis. Notably, PSA has also been detected, albeit at relatively lower levels, in breast cancer [128,129]. This also raises questions about possible actions for PSA-mediated PTHrP cleavage in breast cancer tissues and what function the resulting peptides may have.

### 3.3. Metalloproteases

Metalloproteases are a diverse family of enzymes with functions ranging from cell proliferation to ECM remodeling, due in part to their broad tissue expression and specificities [130]. For example, neprilysin has numerous roles during development based on its ability to inactivate signaling peptides [131,132,133]. Similar to PSA, neprilysin cleaves PTHrP1–34 to generate a 1–23 and 1–26 products [64]. Additionally, neprilysin also cleaves osteostatin (PTHrP107–139) to produce 107–133 and 133–139 fragments [64]. Since amino acids 107–111 comprise the core residues of osteostatin, it is possible that neprilysin generated 107–133 retains activities attributed to osteostatin. Further, the metalloprotease, phosphate regulating gene with homologies to endopeptidases on the X-chromosome (PHEX), has also been shown to cleave osteostatin at three positions near the N-terminal aspartate residues, and this may potentially be responsible for generating PTHrP107–111.

The two most studied sub-classes of metalloproteases are the matrix metalloproteases (MMPs) and the A Disintegrin and Metalloprotases (ADAMs). Although both have been shown to regulate cellular processes by releasing cell surface growth factors [130], the extracellular secretion of MMPs (versus membrane bound ADAMs) greatly expands their access to potential substrates. MMPs are highly expressed in many cancers relative to normal tissues and contribute to invasion and metastasis by cleaving proteins that comprise the extracellular matrix and non-matrix substrates such as cytokines or growth factors thereby regulating their bioactivity. In fact, the non-matrix substrate repertoire for MMPs today outnumbers traditional extracellular matrix substrates [134]. In skeletal malignancies, for example, MMPs have been shown to process many of the factors that drive cancer-bone interactions, including TGF-β, RANKL, and insulin growth factor (IGF) [135,136,137]. PTHrP has also been identified as a novel substrate of MMP-2, -3, -7, -9, and -13, which predominantly generate PTHrP1–17, 18–26, and 27–36 amino acid products [62]. Notably, the novel PTHrP1–17 product retains the ability to signal through PTH1R whereby it induces the osteogenic activities of full length PTHrP1–36, but it does not impact RANKL and osteoclast formation in vivo, even when dosed continuously. The MMP generated PTHrP1–17 could also be detected in conditioned media from prostate cancer and osteosarcoma cells in vitro via mass spectrometry [62]. Given the presumed co-localization of PTHrP with MMPs in cancer, one could hypothesize that PTHrP bioactivity is regulated either directly by MMPs, or through MMP-directed activation of other proteases in the tumor microenvironment. A more complete understanding of the resulting MMP generated PTHrP product activities and specific localization may reveal further insights into PTHrP biology.

### 3.4. Additional Proteases Capable of Cleaving PTHrP

While the majority of studies have defined roles for metalloproteases and serine proteases, other classes of proteases, such as cysteine proteases, are less understood with respect to PTHrP proteolysis but can play roles in post translational modification. For example, prohormone thiol protease (PTP) is a hormone-processing cysteine protease that has been observed to be co-expressed with PTHrP in lung cancer cell lines. In vitro experiments using recombinant proPTHrP1–141 show that PTP cleaves at residue 37 to generate active PTHrP1–36. Interestingly, the locally generated PTHrP was shown to be subsequently involved in regulating cell growth. Furthermore, lung cancer cell lines that express low levels of PTHrP were found not to express PTP [57]. These studies suggest that depending on the tissue context, multiple proteases can contribute to generating PTHrP1–36 and other PTHrP products. 

### 3.5. Biological Considerations for Studying PTHrP Post-Translation Modification

Although evidence suggests that pro-hormones such as PTHrP undergo intracellular proteolytic cleavage in the Golgi prior to secretion of the active protein, PTHrP can also be secreted in longer forms, a finding further supported by the biological detection of PTHrP1–86 for example [71]. Secretion of full length PTHrP, including an intact nuclear localization sequence, increases the likelihood for extracellular proteases to process and regulate PTHrP activity in pathological diseases, either through deregulation/inactivation or generating shorter, bioactive N- and C-terminal products with distinct biological activities.

Of note, the majority of studies to date have focused on these proteolytic events using in vitro models, and while it allows for the identification of whether certain proteases can cleave a selected protein, it does not necessarily confirm that the protein is a substrate in vivo. Spatial localization in tissues is likely key for determining potential cleavage of PTH or PTHrP by these enzymes. For example, PTH is primarily expressed in the parathyroid tissues where furin is also co-expressed [138,139]. However, PC1/3, PC2, and PC5 are not expressed here, and PACE4 is only detected at low levels [116]. In comparison to PTH, PTHrP is expressed in numerous tissues where pro-protein convertases are co-expressed. PC1/3 and PC2 are often found in neuroendocrine tissues, but they are not likely to function extracellularly without the optimal acidic pH profile found in the Golgi. However, like furin, PC5, PC7, and PACE4 are widely expressed in tissues including muscle, heart, brain, intestine, and others. These proteases can also be active at the cell surface, extracellularly, or in the trans Golgi network, but their effects on PTHrP activity in these tissues remains unexplored.

Another limitation of in vitro studies is the inability to include the presence of proteases that may be competing to cleave PTHrP. As discussed, a host of serine proteases, metalloproteases, and cysteine proteases are expressed in similar tissues to PTHrP. Therefore, competition is likely to exist depending upon which protease is more efficient or, spatially, which protease interacts with PTHrP first. For example, in addition to neprilysin, PHEX endopeptidase can process osteostatin (PTHrP 107–139) in 3 positions near the N-terminus of aspartate residues, but the results of the potential interplay of these two enzymes on PTHrP is unknown. Similarly, the detection of PTHrP12–48 from breast cancer patients suggests that the involved proteases are able to produce this form prior to generation of PTHrP1–36, or in a situation where cleavage at phenylalanine 37 does not occur. 

The majority of proteolytic enzymes are secreted in a latent form that requires activation by physiological processes (pH) or by other proteases [140]. Serine proteases and MMPs can reciprocally activate each other. For example, PSA is a potent regulator of MMP-2 activity [141], while conversely, MMPs can activate kallikreins [142]. The timing sequence of these proteolytic cascades could also be important in facilitating the post-translational processing of PTHrP. Furthermore, PTHrP itself has been shown to induce the expression of proteases such as MMPs [141]. This could in turn act as a feedback mechanism to regulate the activity of PTHrP1–36. 

Understanding which proteases process PTHrP and the underlying temporal dynamics of proteolytic post translational modifications may also uncover options that help guide therapeutic intervention for skeletal disorders or malignancies. PTHrP is known to contribute to breast cancer progression as evidenced by studies employing neutralizing antibodies [52]. Although these antibodies were not as effective clinically, they confirmed the potent osteolytic effects of tumor-derived PTHrP. Therefore care should be taken to avoid targeting proteases responsible for the processing of PTHrP1–36. Conversely, understanding the PTHrP products that have anti-resorptive effects or bone anabolic effects such as osteostatin could be beneficial in protecting against cancer-induced osteolysis and/or osteoporosis. However, modulating enzymatic activity in vivo comes with risks, such as off target effects due to their ubiquitous expression. An alternative approach utilizing this knowledge could be the generation of stable PTHrP products that have anti-bone resorptive effects that are modified to prevent their degradation in vivo. Such approaches have already proven beneficial for the treatment of osteoporosis, such as the development and FDA approval of abaloparatide. The abaloparatide sequence shares the first 20 N-terminal amino acids in common with PTHrP, but approximately half of the remaining amino acids are modified to ensure predominantly anabolic properties [44,143].

## 4. Conclusions

PTHrP is known for its control of multiple biological processes in skeletal homeostasis, development, and malignancy. Our knowledge of this multi-functional protein, however, is not yet complete, as additional functional peptide forms of PTHrP generated by proteolytic processing of the full length protein continue to be discovered. While the PTHrP protein sequence offers clues as to potential products and the proteases involved in their genesis, a more precise understanding of the spatio-temporal post translational processing of PTHrP and the biological characterization of the resulting peptides warrants continued investigation. Moving forward, it will be essential to also consider the tissue context of PTHrP processing and the potential proteases therein. The expanding investigations of PTHrP proteolysis and resulting peptide fragments holds potential to uncover new mechanisms for regulating the positive and negative effects of PTHrP on bone and could aid the design of novel therapeutics to tackle bone disease that is commonly associated with skeletal malignancies.

## Figures and Tables

**Table 1:** PTHrP fragments, known functions, and proteases (n/a = not available).

Product	Putative Proteases	Proposed Proteases	Function	Reference
1–139/141/173		n/a	Full Length Protein	
1–36	Prohormone Thiol Protease (PTP), Furin	PC1, 2, 4, 5, PACE4	“Mature PTHrP;” bone homeostasis and developmental roles; ubiquitous tissue expression	[4,18,60]
1–16	Unknown	n/a	Cardiomyocyte contractility; activation of cardiac endothelin-A receptor	[61]
1–17	MMP-2, 3, 7, 9	n/a	Stimulates osteogenesis in vitro and in vivo but does not induce osteolysis; secreted by prostate cancer and osteosarcoma cell lines	[62]
1–23	Neprilysin; Prostate Specific Antigen (PSA)	n/a	Abolished cAMP induction in vitro in mouse osteoblasts; new bone formation in calvaria	[63,64,65]
1–26	Neprilysin	n/a	Unknown	[64]
1–74	Unknown	n/a	Anabolic effects in bone at low dosage in vivo (synthetic peptide)	[66,67]
1–84	Unknown	Trypsin-like or carboxypeptidase B-like enzymes?	Stimulates calcium transport across placenta (in sheep)	[68]
1–86	Unknown	Trypsin-like or carboxypeptidase B-like enzymes?	Promotes osteogenesis and inhibits adipogenesis of human MSCs; positive correlation with humoral hypercalcemia of malignancy; detected in circulation from patients and in breast cancer tissue extracts	[69,70,71,72,73]
1–108	Unknown	n/a	Stimulates calcium transport across placenta (in sheep)	[68]
12–48	Prolyl oligopeptidase	PSA; Dipeptidyl peptidase	Suppression of osteoclast differentiation and survival in vitro; prognostic for bone metastases in breast cancer patient plasma	[4,74,75]
37–67	Unknown	Furin; Prohormone thiol protease (PTP)	Functions unclear; immunoreactivity within amnion covering placenta; Present in pancreatic and breast cancer tissue extracts	[67,69,70,73]
37–74	Unknown	Furin; Prohormone thiol protease (PTP)	Functions unclear; present in plasma from patients with humoral hypercalcemia of malignancy	[67,73]
37–94	Unknown	Furin; Prohormone thiol protease (PTP)	Stimulates calcium transport across placenta (in sheep)	[76]
38–64	Unknown	n/a	Promotes type II lung cell growth and repair	[77]
38–94	Unknown	Peptidyl α-amidating mono-oxygenase	Inhibition of growth, invasion, and viability in breast cancer cell lines in vitro; attenuated breast cancer tumorigenesis in vivo; translocation to nucleoplasm and binding of chromatin; potent activator of intracellular calcium signaling pathway; placental calcium transport; secreted in vitro by RIN cells transfected to express PTHrP	[78,79,80,81]
38–95	Unknown	n/a	Functions unclear; secreted in vitro by RIN cells transfected to express PTHrP	[80]
38–101	Unknown	n/a	Functions unclear; secreted in vitro by RIN cells transfected to express PTHrP	[80]
38–111	Unknown	n/a	Functions unclear; detected in circulation	[82]
67–86	Unknown	Trypsin-like or carboxypeptidase B-like enzymes	Stimulates calcium transport across placenta from maternal to fetal circulation (in sheep); stimulates intracellular calcium and inositol phosphates in human squamous carcinoma cells; inhibit mitogenesis but stimulate metastatic potential of human breast cancer cell line 8701-BC; proapoptotic in type II lung cells	[55,76,83,84,85]
75–84	Unknown	Trypsin-like or carboxypeptidase B-like enzymes	Stimulates calcium transport across placenta from maternal to fetal circulation (in sheep)	[76]
75–86	Unknown	Trypsin-like or carboxypeptidase B-like enzymes	Stimulates calcium transport across placenta from maternal to fetal circulation (in sheep)	[68]
107–111	Unknown	Furin; PHEX	Inhibition of osteoclast resorption in vivo; bone anabolic effects in vivo	[86,87]
107–139	Unknown	Furin	Inhibition of osteoclast resorption in vivo; bone anabolic effects in vivo; osteogenic differentiation in vitro	[88]
107–141	Unknown	Furin	Interacts with cytoplasmic β-arrestin in yeast two-hybrid system (function unclear)	[89]
109–138	Unknown	Furin	Secreted by RIN cells transfected to express PTHrP; elevated levels in detected in plasma from patients with humoral hypercalcemia of malignancy	[90,91]
109–141	Unknown	Furin	Detected in patients with humoral hypercalcemia of malignancy	[71]
